# Giant Cell Tumor of Bone: Documented Progression over 4 Years from Its Origin at the Metaphysis to the Articular Surface

**DOI:** 10.1155/2016/9786925

**Published:** 2016-08-17

**Authors:** Colin Burke, Thomas Link, Richard J. O'Donnell, Soo-Jin Cho, Daria Motamedi

**Affiliations:** ^1^Department of Radiology & Biomedical Imaging, University of California, San Francisco, 505 Parnassus Avenue, M-391, San Francisco, CA 94143-0628, USA; ^2^Department of Orthopaedic Surgery, University of California, San Francisco, 1825 Fourth Street, Fourth Floor, San Francisco, CA 94158, USA; ^3^Department of Pathology, University of California, San Francisco, 1825 Fourth Street, Room M2370, San Francisco, CA 94143, USA

## Abstract

The exact location of origin for giant cell tumors of bone (GCTB) remains controversial, as lesions are not routinely imaged early but rather late when the tumor is large and clinically symptomatic. At the time of diagnosis, GCTB are classically described as lucent, eccentric lesions with nonsclerotic margins, located within the epiphysis to a greater extent than the metaphysis. Here we present a case of a biopsy proven GCTB initially incidentally seen on MRI as a small strictly metaphyseal lesion, which over the course of several years expanded across a closed physis to involve the epiphysis and abut the articular surface/subchondral bone plate.

## 1. Introduction

Giant cell tumors of bone (GCTB) are relatively common tumors, accounting for 5% of all primary bone tumors [1]. GCTB most commonly affect the long bones with 50–65% affecting the knee [1, 2]. Patients with GCTB usually present in their 20 s or 30 s with nonspecific symptoms such as pain, soft tissue swelling, and decreased range of motion at the adjacent joint [3]. The typical radiographic appearance is a lytic lesion without matrix; CT shows a mostly narrow but sometimes wider zone of transition. Margins are usually nonsclerotic and the location is eccentric extending to the subchondral bone. The exact site of origin of GCTB remains controversial, but it is thought that the lesion arises from the metaphyseal side of the epiphyseal plate [1, 4]. However, 84–99% of lesions extend to within 1 cm of articular surface at the time of imaging, making exact localization of the site of origin difficult to assess [4]. We present a unique case of a GCTB that is profiled over 4 years by imaging and shown to arise at the metaphysis and subsequently expand into the epiphysis and, ultimately, abut the articular surface.

## 2. Case Report

**Figure 1:**
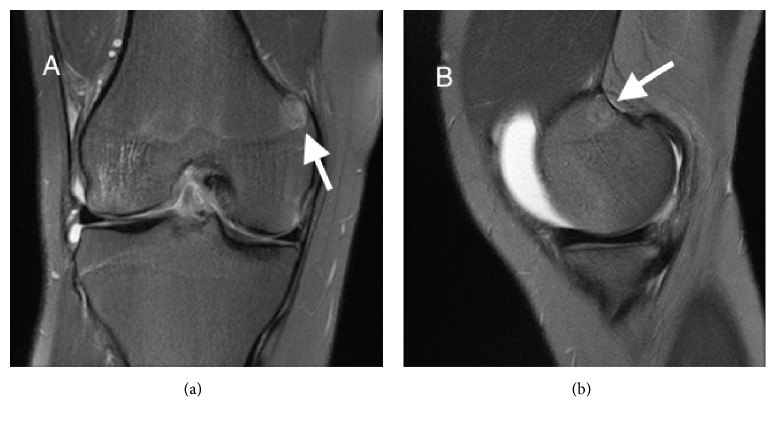
MRI coronal (a) and sagittal (b) T2 fat saturation images at initial presentation for right knee pain status after hyperextension in 2011. The arrows indicate a round T2 heterogeneous but mostly hypointense subcortical intraosseous lesion arising in the posterior aspect of the medial distal femoral metaphysis. There was also a complete ACL tear and knee joint effusion.

A 25-year-old man initially presented to an outside institution in 2011 with a history of a right knee hyperextension injury while playing basketball. He was evaluated with a right knee MRI that showed an anterior cruciate ligament (ACL) tear and a small subcortical lesion at the posterior aspect of the medial metaphysis of the distal femur that was nonspecific and too small to characterize; however, enthesopathy and small cortical based lesions such as nonossifying fibroma, enchondroma, fibrous cortical defect, and fibrous dysplasia were included in the differential diagnosis (Figure 1). He underwent successful ACL reconstruction and recovered well, returning to normal activities. Two years later radiographs were obtained when he developed right knee pain; he was clinically diagnosed with a medial cruciate ligament (MCL) sprain and was referred to physical therapy. His pain continued for several months despite therapy. He switched health insurance coverage and was lost to follow-up but re-presented to our institution in 2015 with persistent right knee pain. Radiographs and an MRI were obtained.

**Figure 2:**
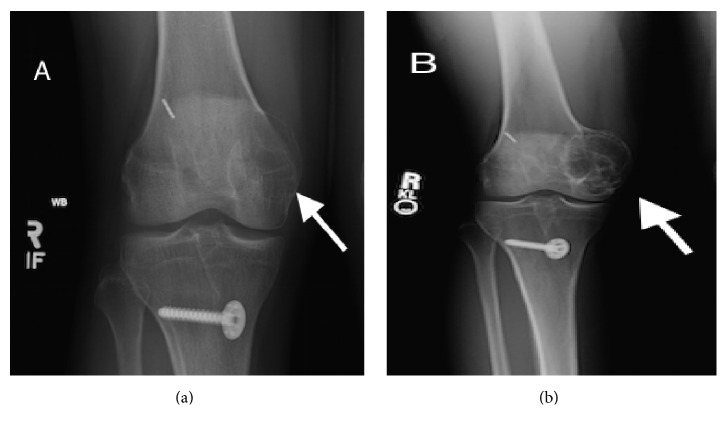
Frontal view radiographs of the right knee obtained (a) 2 years and (b) 4 years after initial imaging demonstrate a closed physis with progressive enlargement of a well-circumscribed, nonsclerotic, eccentric lucent lesion, as indicated by the arrows, at the medial femoral condyle abutting the articular surface.

**Figure 3:**
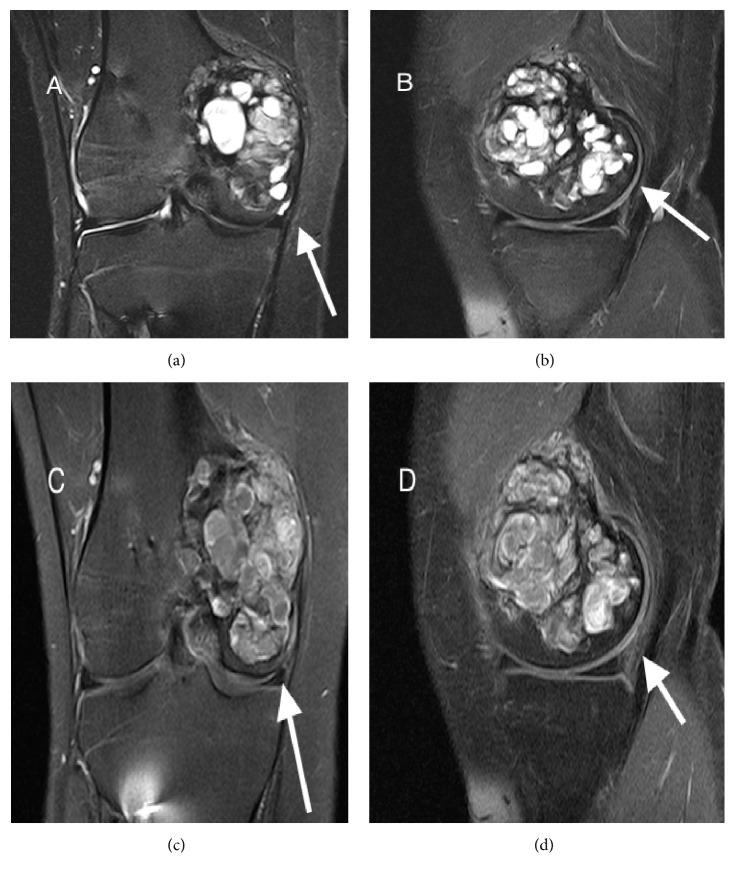
MRI of the right knee 4 years after initial presentation demonstrates an expansile multilobulated mass in the medial femoral condyle. (a) Coronal T2 turbo spin echo (TSE) short tau inversion recovery (STIR), (b) sagittal T2 TSE fat saturation, and (c) coronal and (d) sagittal T1 TSE fat saturation images demonstrating marked interval increase in size of a T1 and T2 heterogeneous multilobulated, expansive osseous lesion, as indicated by the arrows, extending from the posterior distal medial femoral metaphysis to the medial femoral epiphysis. Within this mass are multiple fluid filled spaces with fluid-fluid levels suggestive of intralesional secondary aneurysmal bone cyst formation.

The radiographs obtained in 2013 and 2015, two and four years, respectively, after the metaphyseal lesion was seen on the initial MRI, showed a well-marginated, locally aggressive, eccentric lesion in the right medial femoral condyle extending from the metaphysis to the epiphysis (Figure 2). MRI demonstrated a marked increase in size of this now expansile, multilobulated right medial femoral condylar lesion with fluid-fluid levels extending to the subchondral bone plate (Figure 3).

**Figure 4:**
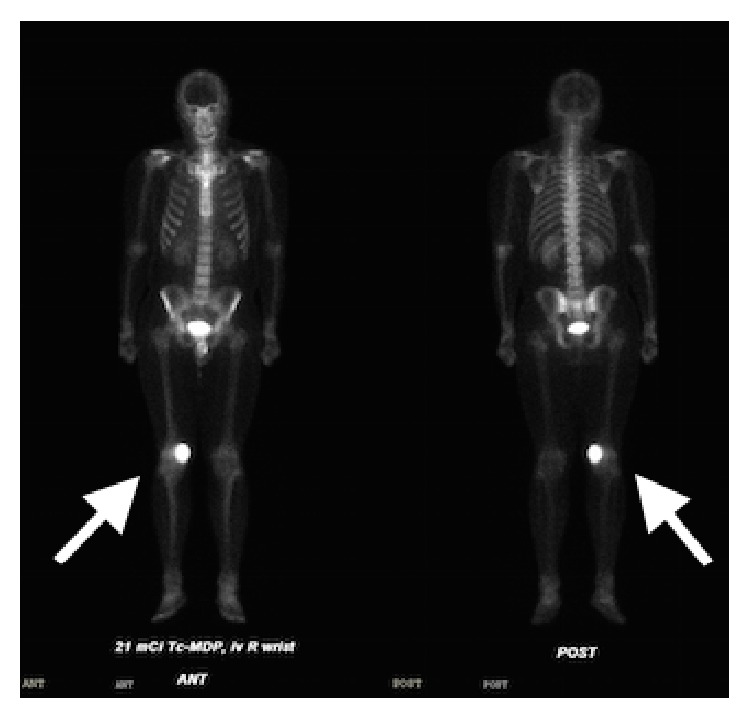
25 mCi technetium total bone scan demonstrating intense uptake in right medial femoral condyle consistent with osseous turnover in the region of the expansile mass, as indicated by the arrows. No other lesions were noted.

Differential diagnosis at this time was more limited and suggestive of GCTB based on age, location, and radiological criteria; however, aneurysmal bone cyst (ABC) and malignant tumors such as telangiectatic osteosarcoma or clear cell chondrosarcoma remained as alternative diagnoses. A total body bone scan was performed, demonstrating intense uptake in the right medial femoral condyle without other lesions identified (Figure 4). The patient was referred to orthopedics in late 2015 and an incisional biopsy and frozen section were performed, followed by immediate curettage, burring, hydrogen peroxide application, argon beam, and cement packing.

**Figure 5:**
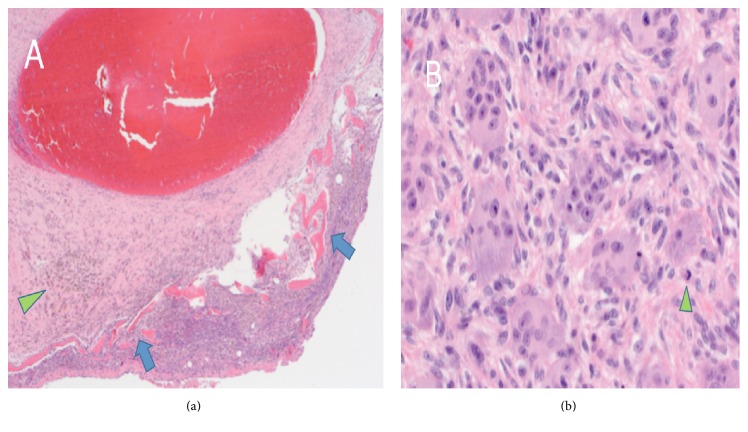
Microscopic examination of curettage specimen. (a) Aneurysmal bone cyst-like area with blood-filled spaces lined by fibrous septa containing reactive bone (blue arrows) and hemosiderin (green arrow head); H&E stain at 40x and at 400x (b) demonstrating giant cell tumor areas with multinucleated giant cells evenly dispersed amongst mononuclear cells with spindled morphology. No overt or diffuse nuclear pleomorphism is identified and while scattered mitotic figures are present (arrow head), no atypical forms are seen.

Macroscopic examination of the curettage specimen showed multiple, irregular fragments of rubbery, red-tan soft tissue, and white-tan hard osteocartilaginous tissue. Microscopic examination revealed blood-filled spaces lined by fibrous septa without an endothelial lining or vascular smooth muscle. The fibrous septa contained scattered multinucleated giant cells, abundant hemosiderin, osteoid matrix or reactive bone, and spindled mononuclear cells with plump nuclei without hyperchromasia or significant increase in mitoses, consistent with an aneurysmal bone cyst (ABC) component (Figure 5(a)). In addition, many areas without fibrosis but with evenly distributed multinucleated giant cells amid plump to spindled mononuclear cells were identified, raising the possibility of GCTB (Figure 5(b)). Fluorescence in situ hybridization (FISH) testing for the* USP6* gene rearrangement, characteristic of primary ABC [5], was negative in this case, further supporting the impression of secondary ABC superimposed on GCTB.

The patient has had an uncomplicated postoperative course: at 6 months after surgery, he has returned to pain-free, full activity, and postoperative radiographs showed no evidence of local recurrence.

## 3. Discussion

GCTB is characterized by the most recent World Health Organization classification as a benign but locally aggressive tumor [6]. Histologically, the tumor consists of numerous osteoclast-like multinucleated giant cells, evenly distributed amongst round to spindled mononuclear cells [1]. Although aggressive intralesional treatment results in an acceptably low local recurrence rate, GCTB can recur locally after surgical excision and can metastasize, most commonly to the lungs [4]. Malignancy has also been described in GCTB, with primary malignancy referring to cases with areas consistent with a high-grade sarcoma present within otherwise conventional GCTB and secondary malignant referring to cases of high-grade sarcoma occurring at the site of previously treated GCTB [6, 7]. GCTB's typical radiographic findings include a well-defined, expansile, and eccentrically located lucent lesion with a narrow zone of transition but a nonsclerotic margin. MRI findings are nonspecific but classically reveal increased signal intensity on fluid sensitive sequences, decreased signal intensity on fat sensitive sequences, and enhancement on postcontrast imaging [1, 8]. Findings are often heterogeneous due to hemosiderin deposition as well as bone and collagen as was noted in our case.

In our patient, initial incidental MRI characterization was nonspecific and showed a small T1 hypointense and T2 heterogeneous subcortical intraosseous metaphyseal lesion. Follow-up radiographs 3 years later displayed the more typical radiographic features described above (Figure 3).

Differential diagnosis included ABC which can be a primary, de novo neoplasm characterized by rearrangements of the* USP6* gene, or secondary, arising in association with other benign or malignant tumors, including GCTB. Primary and secondary ABCs have a similar appearance radiographically and pathologically, and the diagnosis of GCTB with secondary ABC formation is typically confirmed with microscopic evaluation. Radiographically, ABC is more commonly characterized by a sclerotic margin, typically occurs in younger patients compared to GCTB, and often manifests fluid-fluid levels without other soft tissue components [9]. Rare primary bone malignancies such as clear cell chondrosarcoma and giant cell rich osteosarcoma can also mimic GCTB but often have more destructive features, adjacent bone marrow edema, and/or a soft tissue mass component.

Treatment for GCTB is traditionally surgical with curettage and cement packing. However, the local recurrence rate has been reported to be as high as 25% depending on anatomical location [10, 11]. Postoperative radiographs obtained must be closely inspected for new lucencies at the cement bone interface. Recently, the monoclonal antibody drug denosumab has been successfully used to treat GCTB by inhibiting the osteoclastic activity of cells in GCTB. Medical therapy alone with denosumab has been reported to have a dramatic treatment response rate of up to 90% tumor necrosis, with increased sclerosis and reconstitution of cortical bone [12]. This response suggests that denosumab is an effective treatment to reduce tumor size preoperatively, to treat lesions that cannot be treated surgically, or for those patients for whom surgery is not an option.

While GCTB is classically described as abutting an articular surface, its location of origin remains somewhat controversial in the literature. GCTB most often presents in a skeletally mature patient with pain and at the time of initial imaging primarily involves the epiphysis [8]. However, the largest study to date of GCTB in skeletally immature patients described up to 96% of cases to be localized to the metaphysis [13]. The metaphysis is rich in osteoclasts and highly vascularized. Perhaps the increased cell turnover and proliferation, with the resultant increase in possible mutations, explains the metaphyseal origin for GCTB in young adults. Our case report reinforces these findings and the hypothesis that these tumors originate in the metaphysis and migrate towards the epiphysis after closure of the physis. This distinction is important as GCTB should be included in the differential for a metaphyseal lesion to facilitate early diagnosis.

In summary, to the best of our knowledge, this is the first case to clearly demonstrate multiple modalities of the unique progression of a pathologically confirmed GCTB in a single patient over four years from its origin in the metaphysis to its final eccentric location abutting an articular surface.
